# Impact of the COVID-19 Pandemic On Violence Against Children: A Narrative Review

**DOI:** 10.1007/s11920-023-01449-1

**Published:** 2023-09-18

**Authors:** Amera Mojahed, Judith T. Mack, Lina Specht, Vania Sandoz, Susan Garthus-Niegel

**Affiliations:** 1 Institute and Policlinic of Occupational and Social Medicine, Faculty of Medicine, Technische Universität Dresden, Dresden, Germany; 2 Clinical Psychology and Psychotherapy; Institute and Policlinic of Occupational and Social Medicine, Faculty of Medicine, Technische Universität Dresden, Dresden, Germany; 3 Department of Psychotherapy and Psychosomatic Medicine, Faculty of Medicine, Technische Universität Dresden, Dresden, Germany; 4 Child Abuse and Neglect Team, Department Woman-Mother-Child, Lausanne University Hospital, Lausanne, Vaud, Switzerland; 5 Institute for Systems Medicine (ISM) and Faculty of Medicine, Medical School Hamburg, Hamburg, Germany; 6 Department of Childhood and Families, Norwegian Institute of Public Health, Oslo, Norway

**Keywords:** COVID-19 pandemic, Impact, Violence against children, Child marriage, Narrative review, Post-pandemic implications

## Abstract

**Purpose of Review:**

The goal of this paper was to explore the different ways the COVID-19 pandemic has affected violence against children (VAC).

**Recent Findings:**

Recent research of peer-reviewed articles using operational or survey data revealed the pandemic’s impact in terms of institutional responses, risk and mediating factors, changes in VAC dynamics, and a likely increase in child marriage.

**Summary:**

Findings include a decrease in institutional responses, activities, and prevention case openings; an increased incidence of interparental intimate partner violence (IPV) witnessing cases, hospital admissions for suspected Abusive Head Trauma (AHT), other pediatric injuries, and sexual violence; a change in family conflict dynamics; and an estimated increase in child marriages. It also revealed mediating factors between the relationship of the pandemic and VAC (such as parental stress and mental health symptoms), as well as risk factors observed by service providers, which include the risk of mental health symptoms of both parents and children. Post-pandemic VAC research can be improved by utilizing operational or survey data in a meaningful way to be able to derive sound intervention approaches to diminish the pandemic’s impact on VAC and child marriage. We also propose for researchers to integrate child marriage into the definition of VAC.

## Introduction

The COVID-19 pandemic could have had far-reaching consequences for various aspects of society, including the well-being and safety of children. Following the United Nations Convention on the Rights of the Child, violence against children (VAC) is the abuse and neglect that occurs to children under 18 years of age. It includes all types of physical and/or emotional ill-treatment, sexual abuse, neglect, witnessing violence, commercial, or other exploitation that results in actual or potential harm to the child’s health, survival, development, or dignity in the context of a relationship of responsibility, trust, or power [1].

Prevalence rates of VAC vary considerably across countries and research methods [1]. These rates are influenced by the various definitions of VAC, its types, the extent and reliability of official statistics, and the coverage and quality of surveys that rely on different sources of information, e.g., self-reporting by victims, parents or caregivers [2]. Pre-pandemic studies indicate large gaps in existing global data on the prevalence and perpetrators of different forms of VAC in different ages. Age-specific and sex-specific data on witnessing interparental intimate partner violence (IPV) are also rare [2].

Despite these gaps, a most recent pre-pandemic meta-regression revealed prevalence rates of physical and psychological violence, neglect, and witnessing interparental IPV for both boys and girls from the age of two to fourteen years to be greater than 50% [2]. According to a recent review, the rates of physical, psychological, and sexual abuse and neglect during the COVID-19 pandemic between the years 2020 and 2022 ranged from 0.1%–71.2%, 4.9%–61.8%, 1.4%–19.5%, and 7.3%–40%, respectively [3]. The review also found a decline in the reporting of VAC, yet an increase in severe cases of child maltreatment during the COVID-19 pandemic compared to pre-pandemic rates [3].

The pandemic has brought to light several risk factors contributing to a potential increase in physical and psychological VAC that are likely to vary by setting and population. Concerns have been raised about the increased vulnerability of children in many households as a result of stay-at-home orders, disruption of regular routines, school closures and reduced access to support services [4], as well as pre-existing inequalities, and a greater amount of economic stressors, such as job loss, financial instability, and food insecurity [3–7].

According to the latest United Nations Children’s Fund’s guideline on research on VAC during the COVID-19 pandemic for generating evidence [8], researchers are encouraged to adopt a holistic approach in generating actionable evidence. While understanding VAC levels is essential, it is equally important to consider additional types of information and indicators that can provide insight into how best to address the issue. This entails examining risk factors that contribute to VAC, evaluating the accessibility of services for those affected, and assessing the impacts of interventions designed to mitigate and prevent VAC.

The guideline highlighted the importance of identifying the sources used for data collection as a crucial first step [8]. This is particularly critical because the type of data sources may answer different questions that in turn are used to inform advocacy and action related to VAC during the COVID-19 pandemic [8]. These sources can include a wide range of channels through which data on VAC can be collected, such as from the children themselves or their parents or primary caregivers (known as survey data), or from systems or individuals who deal with the affected (known as operational data). Each of these sources has unique strengths and limitations that need to be carefully considered to ensure reliable and comprehensive data collection.

Therefore, and in an attempt to obtain a more holistic understanding of the subject, we will first review the included studies according to the main types of data on VAC that were used. Secondly, we will identify the ways in which COVID-19 has impacted such violence by exploring frequency of service responses, mediating effects of already known risk factors, and changes in abuse dynamics. Therefore, our aim was to critically evaluate the literature on the impact of the COVID-19 pandemic on VAC perpetrated by parents or other primary caregivers and co-occurring IPV, if present.

## Methods

We searched PubMed, Embase, and PsycINFO for peer-reviewed articles using the terms ‘violence against children’, ‘child maltreatment’, ‘child abuse’, ‘child neglect’, ‘child exposure to intimate partner violence/domestic violence’, ‘violent discipline’, ‘corporal punishment’, ‘child sexual abuse’, and ‘sexual exploitation’ as search terms for violence (Appendix A). We included English, German, and French articles published between 2019 and 2023.

The screening involved two independent reviewers and data extraction was performed by both reviewers for 50% of the studies. Disagreements were resolved by a third reviewer. The studies that fulfilled our inclusion criteria are depicted in Table 1. Following the UN’s categorization of VAC (1), we decided to focus on the following types: Maltreatment (i.e., physical, sexual, and psychological violence, as well as neglect) by parents or primary caregivers and witnessing interparental IPV.

**Table 1:** Inclusion and exclusion criteria

**Inclusion criteria**	**Exclusion criteria**
Children and adolescents under 18 years of age	Individuals older than 18 years of age
Violence against children: maltreatment (physical, sexual, psychological, neglect), and witnessing interparental IPV	Other types of VAC
Perpetrators: parents or primary caregivers (such as step parents or adoptive parents)	Perpetrators other than the parents or primary caregivers, e.g., siblings, school peers, or intimate partners, as the sole perpetrators
Peer-reviewed articles, quantitative, qualitative, observational studies	Gray literature, reviews, editorial-like and discussion papers

## Results

**Fig. 1:**
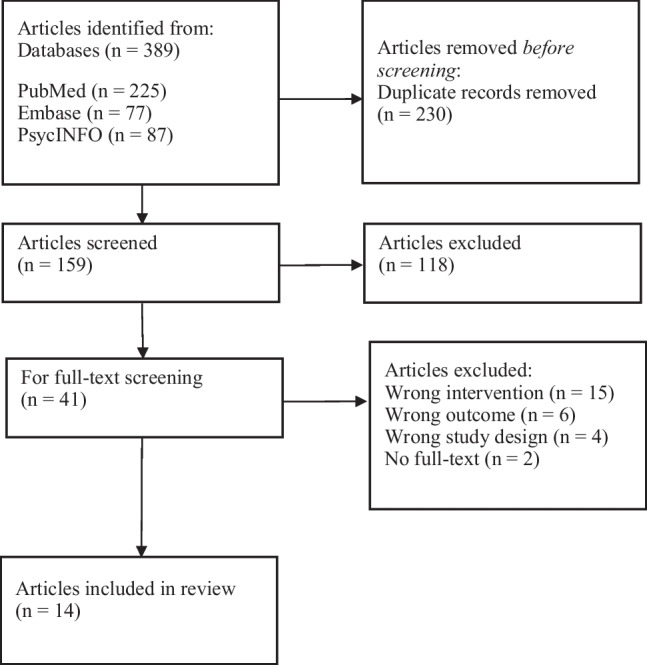
Flow diagram

A total of 14 published articles were included (see Fig. 1) [9–13, 14•, 15••, 16, 17•, 18••, 19•, 20•, 21•, 22••]. Studies were reviewed in two main overarching sections: Operational data (A) and survey data (B) (Table 2).

**Table 2:** Extraction table for included studies

**Reference**	**N**	**Exact age group**	**Violence label (types)**	**Topic of Review**
**Operational data**				
Brown et al. [9]	from 12,329 to 9,386 responses	-	Child maltreatment (sexual abuse, domestic violence, physical abuse, and neglect)	Frequency of CPS responses
Bullinger et al. [10]	- serve parents in urban clusters/suburbs (n = 121, 46.9%) - serve urban areas (n = 63, 25%) - serve rural areas (n = 65, 26%)	-	Family violence or child maltreatment ((1) aggressive conflict between adult members of the household, (2) child emotional/verbal abuse, (3) children being left unsupervised, (4) intimate partner violence, (5) child physical or medical neglect, (6) child physical abuse, and (7) child sexual abuse)	Risk factors reported by service providers
Focardi et al. [14•]	167	-	Witnessing interparental IPV	Children witnessing interparental IPV
Massiot et al. [16]	1583	≤ 15 years	Child abuse and neglect	Activity of CAC
Sinko et al. [19•]	105	10 – 18	Family conflict and abuse	Change in family conflict dynamics
Whaling et al. [21•]	-	-	Family conflict and abuse	Frequency of prevention case openings
**Survey data**				
Caron et al. [11]	3452	< 2 years	Child abuse (AHT, SDH)	AHT and other pediatric injuries
Cercone et al. [12]	82	< 5 years	Child abuse (skull fractures (with) intracranial injury; abnormal skeletal survey; retinal hemorrhages; abnormal cervical spine imaging; SDH)	AHT and other pediatric injuries
Sethuraman et al. [18••]	3130	≤ 21 years	Child physical abuse (CPA-related injuries: head trauma, fractures, bruises/patterned injury, thoracoabdominal injury, oral trauma)	AHT and other pediatric injuries
Pannizzotto et al. [17•]	67	< 16	Child maltreatment (physical, sexual, psychological, and serious neglect)	Sexual violence
**General survey data**				
Chung et al. [13]	258	0 – 12	Harsh parenting	Mediating factors and covariates
Liu et al. [15••]	4692	10 – 18	Child maltreatment (psychological and physical violence)	Mediating factors and covariates
Tso et al. [20•]	417	2 – 12	Child maltreatment (physical assault, psychological aggression, neglect, non-violent discipline)	Children with vulnerabilities
Yukich et al. [22••]	-	< 18	Child marriage	Child marriage

*CPS* Child Protective Service, *CAC* Child Advocacy Center, *AHT* Abuse Ahead tTrauma, SDH Subdural Hemorrhage, *CPA* Child Physical Abuse

### Operational Data

Here, included studies explored frequency of child protective service (CPS) responses, which includes receiving and screening reports or referrals of suspected VAC, conducting comprehensive investigations, providing interventions and ongoing monitoring of the child's safety and welfare [9], activity of child advocacy centers (CAC) [16], or frequency of prevention case openings [21•]. Service provision data (i.e., data collected from service providers) were used in three studies [10, 14•, 19•] the incidence of children witnessing interparental IPV [14•], and a change in family conflict dynamics [19•]. Four other studies utilized hospital records and presented changes in incidence of hospital admissions for Abusive Head Trauma (AHT) [11, 12] or other pediatric injuries [18••], and sexual violence [17•].

#### Frequency of CPS Responses

A study by Brown et al. revealed an overall decline in the number of referrals and frequency of CPS responses during the pandemic [9].

#### Activity of CAC

Massiot et al. indicated a decrease in consultations activity of CACs and judicial activities during the lockdown [16].

#### Frequency of Prevention Case Openings

A study by Whaling et al. (2023) determined whether families were receiving preventive services when parents and their children were indefinitely isolated from the outside world. The goal of prevention case openings was to promote the safety and welfare of the child while maintaining family unity whenever possible. Indeed, opening a new VAC prevention service case during quarantine declined by 49.17% [21•].

#### Risk Factors Reported By Service Providers

In a study evaluating VAC and family violence risk during the COVID-19 pandemic using an evidence-based telehealth home visiting program, service providers reported that caregivers who struggled to maintain social distance due to their employment and continued to work outside the home were more likely to report an increase in children being left unsupervised and to physically or medically neglect their children [10].

They also indicated that family members with heightened anxiety or nervousness were more likely to report increased child psychological abuse. Providers who reported that the children or caregivers they looked after seemed more frustrated than usual also reported a perceived increase in the frequency of aggressive conflicts between adults and physical or medical neglect of children. Providers’ reports of families accessing public benefits, as an indicator of low socio-economic status, were not consistently related to providers’ perception of heightened VAC [10].

#### Children Witnessing Interparental IPV

The study by Focardi et al. (2022), which utilized medical records, highlighted that the witnessed abuse was interparental IPV in most cases, accounting for the majority 79% of the reported cases [14•]. Among these interparental IPV incidents, 74% consisted of physical violence. The study also found that in 12% of the cases, minors themselves became victims of physical abuse. No statistically significant relationships were found between the beginning of the COVID-19 pandemic and the changes in the number of cases of domestic abuse. Of the children affected, 49% belonged to an ethnic minority.

#### Change in Family Conflict Dynamics

A study by Sinko et al. (2022) looked into how COVID-19 has impacted family conflict and abuse from the perspectives of children who accessed a national child abuse hotline that serves the U.S. and Canada [19•]. Many family conflicts emerged because of children's reduced productivity in school or due to not having done their chores during stay-at-home orders.

Children often voiced feeling unable to find relief from family conflict. This was exacerbated by physical distance from alternative social support networks and limited contact with typical safe places (e.g., school, sports, and other afterschool activities) or supportive adults, such as relatives, school counselors, doctors, and coaches. Technological isolation was also reported either as punishment or to keep children from contacting those outside the home. Children reported parents breaking their phones or taking them away. Some children kept their phones secretly [19•].

#### AHT and Other Pediatric Injuries

One study found no significant differences in the number of hospital admissions for AHT [11]. Another study showed that the mortality rate among children with AHT was higher during the pandemic [12].

Sethuraman et al. (2021) reported a significantly greater incidence of emergency room visits related to suspected child physical abuse, dog bites, and firearms [18••]. It also revealed significantly increased incidences in trauma alerts, injury severity, critical care admissions, and deaths. An overall reduction in trauma-related emergency room visits was reported. However, the proportion of injury visits secondary to suspected child physical abuse, neglect, and firearms increased and the mean age was lower.

#### Sexual Violence

Sexual violence emerged as the most frequent form of abuse in hospital-admitted cases, especially among girls (62.5%) and in the age group three to six years, a much higher percentage compared to previous years [17•]. In 50% of these cases, identifying 'disclosure' meant taking the child's word, or observing hyper-sexualized behaviors in the school environment when classes were resumed [17•]. Other disclosures of sexual abuse were made through complaints or requests for care by one of the parents following children’s disclosure of the abuse [17•]. In one case, the diagnosis of gonorrhea in an 8.5-year-old patient was the starting point for further treatment. As far as the perpetrators were concerned, 100% were suspected to be part of the child's close circle, and to have lived in the same household at the time of the events [17•].

### Survey Data

In four studies, VAC was reported by either a parent or a child. Two of them investigated mediating factors in the relationship between the pandemic and the violence [13, 15••, 20•, 22••].

#### Mediating Factors and Covariates

The data reported by Chung et al. (2022), using parental reports, indicated parenting stress to be a significant mediator in the relationship between the perceived impact of COVID-19 and harsh parenting [13]. Parenting stress was defined as “*a psychological reaction when parents experience parental demands that are inconsistent with expectations, or when the parents do not have the resources to meet these demands”* (p. 802), and harsh parenting was defined as “*coercive, aggressive, and emotionally charged disciplinary practices such as caning, spanking, yelling, or shouting at children”* (p. 803).

A further study based on children’s reports showed that the mediating role of child mental health significantly affected the associations between child psychological abuse and suicidal ideation and suicidal behaviors, respectively [15••]. Multiple mediation effects between COVID-19 impact (i.e., job loss of parents and of adolescents and school closures) as well as adolescent suicidal measures were observed, e.g., parental job loss → psychological abuse of children → children’s poor mental health status → suicidal ideation and behaviors.

With respect to covarying factors, children’s sexual orientation (i.e., non-heterosexual male adolescents) was the most consistent and highest risk factor for suicidal intention and behaviors [15••]. Meanwhile, better family and school relations possibly contributed to a reduced probability of having suicidal intention.

#### Children with Vulnerabilities

One study revealed that children with neurodevelopmental disabilities were at higher risk of severe physical assault compared to typically developing children during the COVID-19 pandemic. They also presented with poorer mental health [20•].

#### Child Marriage

Child marriage refers to any formal marriage or informal union (i.e., illegal marriages not sanctioned by the state, usually through religious ceremonies) between a child under the age of 18 years and an adult or another child [23]. One study investigated the potential effects of the pandemic on child marriage prevalence in the five countries that together account for approximately 50% of the world’s child marriages (i.e., Bangladesh, Brazil, Ethiopia, India, and Nigeria) [22••].

It was found that the total number of excess child marriages could range from 3.5 million to 4.9 million in the unmitigated scenario and from 1.8 million to 2.7 million in the mitigated scenario [22••]. In the unmitigated scenario, no specific actions or programs are anticipated to be implemented to reduce the impact of the COVID-19 pandemic on child marriages due to the imposed restrictions, making it in a sense a best estimate of a worst-case scenario. This scenario also assumes that although the pandemic may be short-lived, the effects of the economic and social shocks may persist. In contrast, the mitigated scenario adds specific actions and/or programs that are anticipated to be implemented to reduce the impact of the COVID-19 pandemic on child marriages [22••].

In an unmitigated scenario, up to 10 million marriages for girls can be expected due to the pandemic. In addition to these different scenarios, the estimates are based on theorizing an elevated risk to the five pathways through which an elevated marriage hazard is expected in the context of the COVID-19 pandemic, i.e., death of a parent, interruption of education, pregnancy risk, household income shocks, and reduced access to programs and services [22••].

## Discussion

We reviewed the impact of the COVID-19 pandemic on VAC from studies that utilized operational and survey data. The discussion of findings will be in relation to institutional responses, risk and mediating factors, and changes in abuse dynamics.

### Institutional Responses

An observed effect of the pandemic has been the decline in protective services responses, judicial activities, and the opening of prevention cases related to VAC. These declines suggest the presence of potential barriers to identifying and addressing instances of VAC. For instance, limited access to traditional mandated reporters of VAC, such as daycare and school professionals, as well as healthcare workers, may have occurred [24].

Additionally, reduced availability of telehealth technology due to physical (e.g., lack of access for disadvantaged families and limited access to confidential and safe space) and therapeutic limitations (e.g., challenges in assessing and treating severe clinical presentations) may have further hindered the reporting and prevention of VAC [25]. Moreover, reduced access to VAC prevention services during the pandemic has raised concerns about an increased risk of VAC and, as a result, an elevated number of out-of-home removals (i.e., the legal and social process of removing a child from their home due to concerns for their safety, well-being, or inadequate care. It involves placing the child under the care and supervision of an alternative caregiver or in a residential facility outside their own home) [26].

### Risk and Mediating Factors

Risk factors reported by service providers during the pandemic were more related to mental health symptoms, such as anxiety, nervousness, and frustration of both parents and children, rather than the socio-economic status of the parents or primary caregivers. This could emphasize the role of the COVID-19 pandemic in aggravating pandemic-related parental stress, leading to a significant rise in conflicts within the household [27, 28]. Parental stress was also found to be a mediator between the perceived impact of COVID-19 and harsh parenting.

Further, we found that economic strains caused by parental and minors’ job loss could elevate the risk of child abuse and suicidal behaviors in minors. This finding is consistent with previous studies, showing that physical isolation and unemployment due to the pandemic were positively linked to parent–child conflict and child abuse [4]. A further finding demonstrated that non-heterosexual children were at much higher risk for conducting suicidal behaviors compared to their heterosexual peers, which also aligns with previous studies [29]. This finding may have implications for how the pandemic affects children's experiences of violence and their emotional and psychological distress, especially when factors such as their social or sexual identity are considered.

### Changes in Abuse Dynamics

Family conflicts during the pandemic often involved topics such as children's lack of productivity at school. In addition, children most often identified a lack of technological connectivity and limited social support as reasons for their increased emotional distress and inability to find relief. In some cases, parents' control of children’s phone use made it particularly difficult for children to report to child abuse hotlines, limiting their ability to seek professional interventions. This could be of particular concern seeing as any type of isolation can be a powerful control and abuse tactic [30].

Witnessing interparental IPV is recognized as a form of psychological child abuse [31–34]. From a legal perspective, both European Union treaties, such as the Istanbul Convention [35], and national laws in numerous countries (e.g., Australia, Canada, Germany, Tunisia, Switzerland, UK, and USA) recognize children who witness interparental IPV as victims of abuse [33]. In fact, in one included study, the majority of reported cases (79%) occurred at home, compared with global pre-pandemic rates of more than 50% [2]. Early identification of witnessing interparental IPV is crucial in order to mitigate negative outcomes, such as internalizing and externalizing disorders [36]. However, the COVID-19 pandemic may have limited the access to necessary services and disclosing witnessed interparental IPV episodes, leading to delays in interventions.

In this review, we also examined the findings regarding the rise in mortality among children with suspected AHT, dog bites, and firearm-related injuries during the COVID-19 pandemic. This increase in mortality could potentially be attributed to a genuine rise in fatalities resulting from VAC. However, it can also be attributed to other reasons, and the potential for misdiagnosis of such injuries should not be overlooked [37]. Additionally, the review identified a higher prevalence of sexual violence among cases admitted to hospitals, which aligns with similar survey-based findings reported during the pandemic [38].

A pre-pandemic study conducted by Chan et al. (2016) found that children with disabilities were 1.6 times more likely to experience physical maltreatment throughout their lifetime. Families raising children with neurodevelopmental disabilities could have faced additional stressors compared to families with typically developing children [39]. For example, children with neurodevelopmental disabilities more often experience adverse mental health effects, including higher rates of anxiety and depression [40]. The pandemic has exacerbated the challenges faced by these families, as the reduced availability of social support networks from schools and rehabilitation centers has further strained their situation [41].

In general, criminological theories can further identify some of the pathways that may have led towards the perpetration of VAC [42]. Finkelhor and Asdigian’s targeted congruence theory presents three explanatory factors for the increase in VAC: (a) the vulnerability of potential victims, due to characteristics of the context or of the victims themselves that increase the likelihood of victimization, such as their dependence on an adult, their physical weakness, and their greater social isolation; (b) the *satisfaction* generated by the use of violence, whether it be sexual, as in the case of sexual abuse, or as a way of discharging tension in the use of physical and emotional violence; and (c) antagonism, linked to characteristics or attributes of the child that arouse impulses of rejection or violence in the victimizer, such as constant requests for attention and care [43]. Indeed, these factors or mechanisms could converge and interact with the early-mentioned stressors triggered or exacerbated by the COVID-19 pandemic [44].

Though it is not typically included in the mostly used definitions of VAC, child marriage is a form of violence for several reasons. It typically involves a lack of informed consent from the child. Children are not capable of making mature decisions about marriage, and they may be coerced, forced, or deceived into marriage against their will [23]. Child marriage often stems from harmful traditional practices, social norms, and gender inequality. It perpetuates the cycle of discrimination against girls and reinforces gender-based violence [45].

Moreover, female children often face significant health risks due to early pregnancy and childbirth. Their bodies are not fully developed, making them more vulnerable to complications during pregnancy, childbirth, and maternal mortality. Additionally, child brides are more likely to experience domestic violence and sexual abuse within the marriage [46]. Child marriage disrupts male and female children’s education and denies them opportunities for personal growth, skill development, and economic independence. It perpetuates the cycle of poverty and limits their future prospects [23, 47].

Moreover, child marriage is not a problem confined to low-income countries; it also occurs in high-income countries. In the UK, rates of forced marriage are increasing, with a recent government report highlighting that 33% of forced marriages occur before the age of 18 [48]. Global efforts need to recognize child marriage as a violation of human rights and a harmful practice that perpetuates gender inequality and VAC. Today, multiple crises – including political conflict, climate shocks, and the ongoing fallout from COVID-19 – are threatening to reverse progress towards eliminating these human rights violations.

From a cross-cultural perspective, the impact of COVID-19 may have also taken place in other countries where child marriage is prevalent, such as Yemen and Sudan, where political unrest and armed conflicts are present. In Jordan, for example, national data from the year 2020 related to child marriage shows a slight increase [23]. During the pandemic, the simplicity and lower cost of marriage procedures became increasingly attractive, especially as more families struggled with the adverse economic effects of the crisis. As a result, child marriage emerged as a negative coping mechanism in such circumstances, potentially fuelling the observed increase [49]. Prior to the pandemic, the financial burden associated with marriage may have played an important role in discouraging young men from entering into marriage [49]. The simplicity of marriage procedures was assumed, since the increase in early marriages occurred despite the Supreme Judge Department issuing strict instructions for the granting of permission of the marriage of minors [49]. In Sudan, lack of income and interruption of education are the main factors contributing to an increase of child marriage during the pandemic [23].

In general, it can be expected that child marriage rates will increase in the aftermath of the COVID-19 pandemic. One factor contributing is the poverty or destitution that is prevalent in the countries studied. The economic consequences of the pandemic have led to even greater needs and desperation. Another important factor is education. Following the pandemic, access to education has been restricted, making it unavailable to many families. The lack of resources such as internet access, telephones, and a suitable learning environment exacerbates the problem. As a result, there has been an alarming increase in the number of school drop-outs which significantly increases the risk of child marriage [23].

### Limitations of Existing Studies

Operational data such as administrative records can be imprecise when trying to determine the magnitude of the problem. They are typically limited to the monthly count of known cases, and do not take into account factors such as seasonal fluctuations and underreporting of cases that could not be detected [50]. Further, administrative data related to pediatric injuries can be misleading when it comes to determining whether they are a direct consequence of VAC or merely a suspicion. This can lead to an overestimation of VAC cases, potentially leading to a misallocation of resources and harm to the families served.

It is important to exercise caution and conduct thorough investigations to differentiate between actual cases of VAC and other potential causes of pediatric injuries. This helps ensure that resources are directed towards genuine cases and interventions that are most needed. Survey data on the other hand employ different definitions in different contexts. This makes comparability difficult, which is important in order to make the issue relevant on a larger scale where it could influence policy making and national legislation.

### Implications for Practice and Research

VAC interventions and prevention activities.

1. In terms of child witnessing interparental IPV, a common concern should be an improved integration of the protection of women against violence with child protection in cases of violence where co-victimization occurs [51].
2. Self-report studies on experiences of VAC in general, and sexual child abuse in particular are scarce, and are not directly comparable to studies using helpline data or data from emergency departments. This calls for more research using survey data, as it is well-known that disclosures of violence and sexual abuse are delayed and often go undetected for several years [52].

### Child marriage Prevention Activities

1. Ensuring continued post-pandemic access to education, implementing effective legislation, ensuring access to health and social services, including sexual and reproductive health services.
2. Providing comprehensive social protection measures for families, with particular attention to families that have lost their breadwinner during social crises such as the pandemic, e.g., by establishing social benefit programs for economically disadvantaged families.
3. Include child marriage in the global definitions of VAC. For instance, many unknown cases of informal unions could be identified in high-income countries where there is little to no previous data on the issue. This, in turn, could improve the health and well-being of the many people affected, as their experience of child marriage is now addressed and seen as a form of VAC. Despite comparatively higher levels of access to mental health services in many high-income settings, the secretive nature of child marriage makes it highly unlikely that those in need of support for related mental health issues will seek help [53].

## Conclusions

The ways in which COVID-19 has impacted VAC are numerous. They include a decrease in responses of institutions and prevention case openings; increased incidences of interparental IPV witnessing cases; hospital admissions for suspected AHT and other pediatric injuries; sexual violence; change in family conflict dynamics; and an estimated increase in child marriages. Our narrative review also revealed mediating factors between the relationship of COVID-19 and VAC (such as parental stress and mental health symptoms), as well as risk factors observed by service providers, which include the risk of mental health symptoms of both parents and children.

A good starting point to improve post-pandemic VAC research is to utilize operational or survey data in a meaningful way to be able to derive sound actions and intervention approaches to reduce VAC as well as to integrate child marriage into global definitions of VAC used in research. Practical implications of this review suggest integrating the protection of women and children from violence and ensuring continued access to education after the pandemic, implementing effective laws and policies, and ensuring access to health and social services in the case of child marriage.

## Appendix A: Search Strategy

**PubMed:**

P: ('newborn'[Text Word] OR 'infant'[Text Word] OR 'neonate'[Text Word] OR 'baby'[Text Word] OR 'child'[Text Word] OR 'kid'[Text Word] OR 'adolescent'[Text Word] OR 'youth'[Text Word] OR 'juvenile'[Text Word] OR 'boy'[Text Word] OR 'girl'[Text Word] OR 'teen'[Text Word] OR 'toddler'[Text Word] OR 'preschool'[Text Word] OR ' pupil'[Text Word] OR 'pediatrics'[Text Word] OR 'minor'[Text Word] OR 'underage'[Text Word] OR 'young'[Text Word])

I: (impact[Text Word]) AND ((((coronavirus[Text Word] OR corona-virus[Text Word]) AND (wuhan[Text Word] OR beijing[Text Word] OR shanghai[Text Word] OR Italy[Text Word] OR South-Korea[Text Word] OR korea[Text Word] OR China[Text Word] OR Chinese[Text Word] OR 2019-nCoV[Text Word] OR nCoV[Text Word] OR COVID-19[Text Word] OR Covid19[Text Word] OR SARS-CoV*[Text Word] OR SARSCov2[Text Word] OR ncov[Text Word])) OR (pneumonia[Text Word] AND Wuhan[Text Word]) OR "COVID-19"[Text Word] OR "2019-nCoV"[Text Word] OR "SARS-CoV"[Text Word] OR SARSCOV2[Text Word] OR 2019-nCov[Text Word] OR "2019 coronavirus"[Text Word] OR "2019 corona virus"[Text Word] OR covid19[Text Word] OR ncov[Text Word] OR "novel corona virus"[Text Word] OR "new corona virus"[Text Word] OR "nouveau corona virus"[Text Word] OR "novel coronavirus"[Text Word] OR "new coronavirus"[Text Word] OR "nouveau coronavirus"[Text Word] OR "Long Covid"[Text Word] OR "Long Covid"[Text Word])

I: (impact and COVID-19 pandemic).tw.

O: (child abuse or child maltreatment or violence against children or child neglect or violent discipline or corporal punishment or child sexual abuse or sexual exploitation or mother treated violence or witnessing violence).tw.

**PsycINFO:**

P: (TX newborn) OR (TX neonat*) OR (DE "Neonatal Period") OR (TX baby) OR (TX toddler) OR (TX kindergartner*) OR (TX kindergartener*) OR (DE "Kindergarten Students") OR (TX preschool*) OR (TX pre-school*) OR (DE "Preschool Students") OR (DE "Nursery School Students") OR (TX child*) OR (TX schoolchild*) OR (TX school?child*) OR (TX schoolkid*) OR (TX school?kid*) OR (TX pupil*) OR (TX student) OR (DE "Students") OR (DE "Middle School Students") OR (DE "Primary School Students") OR (DE "Junior High School Students") OR (DE "Intermediate School Students") OR (DE "High School Students") OR (DE "Elementary School Students") OR (TX preteen*) OR (TX pre?teen*) OR (DE "Early Adolescence") OR (TX teen*) OR (TX adolescen*) OR (TX puberty) OR (DE "Puberty") OR (TX pubescent*) OR (TX youth*) OR (TX young*) OR (TX juvenile) OR (TX child*) OR (TX kid*) OR (TX boy*) OR (TX girl*) OR (TX pediatric*) OR (TX paediatric*) OR (TX minor*) OR (TX underage*)

I: TX impact AND TX covid-19 pandemic

O: TX child abuse OR TX child maltreatment OR TX violence against children OR TX child neglect OR TX violent discipline OR TX corporal punishment OR TX child sexual abuse OR TX sexual exploitation OR TX mother treated violence OR TXwitnessing violence

Filter applied in all data banks: 2019 – 2023

## References

[1] WHO. INSPIRE Handbook Action for implementing the seven strategies for ending violence against children. Geneva. 2018.

[2] Devries K, Knight L, Petzold M, Merrill KG, Maxwell L, Williams A, et al. Who perpetrates violence against children? A systematic analysis of age-specific and sex-specific data. BMJ Paediatr Open. 2018;2:e000180. 10.1136/bmjpo-2017-000180. 10.1136/bmjpo-2017-000180 PMC5842994 29637183

[3] Huang N, Yang F, Liu X, Bai Y, Guo J, Riem MME. The prevalences, changes, and related factors of child maltreatment during the COVID-19 pandemic: A systematic review. Child Abuse Negl. 2023;135:105992. 10.1016/j.chiabu.2022.105992. 10.1016/j.chiabu.2022.105992 PMC9755012 36549089

[4] Griffith AK (2022). Parental Burnout and Child Maltreatment During the COVID-19 Pandemic. J Fam Violence.

[5] Wu Q, Xu Y (2020). Parenting stress and risk of child maltreatment during the COVID-19 pandemic: A family stress theory-informed perspective. Developmental Child Welfare.

[6] Koerber MI, Mack JT, Seefeld L, et al. Psychosocial work stress and parent-child bonding during the COVID-19 pandemic: clarifying the role of parental symptoms of depression and aggressiveness. BMC Public Health. 2023;23(1):113. Published 2023 Jan 16. 10.1186/s12889-022-14759-5. 10.1186/s12889-022-14759-5 PMC9841494 36647046

[7] Brym S, Mack JT, Weise V, Kopp M, Steudte-Schmiedgen S, Garthus-Niegel S. Mental health of working parents during the COVID-19 pandemic: can resilience buffer the impact of psychosocial work stress on depressive symptoms?. BMC Public Health. 2022;22(1):2426. Published 2022 Dec 26. 10.1186/s12889-022-14582-y. 10.1186/s12889-022-14582-y PMC9790816 36567325

[8] United Nations Children’s Fund (2020). Research on Violence against Children during the COVID-19 Pandemic: Guidance to inform ethical data collection and evidence generation.

[9] Brown SM, Orsi R, Chen PCB, Everson CL, Fluke J (2022). The Impact of the COVID-19 Pandemic on Child Protection System Referrals and Responses in Colorado, USA. Child Maltreat.

[10] Bullinger LR, Marcus S, Reuben K, Whitaker D, Self-Brown S (2022). Evaluating child maltreatment and family violence risk during the COVID-19 Pandemic: Using a telehealth home visiting program as a conduit to families. Infant Ment Health J.

[11] Caron F, Tourneux P, Tchidjou HK, et al. Incidence of child abuse with subdural hemorrhage during the first year of the COVID-19 pandemic: a nationwide study in France [published correction appears in Eur J Pediatr. 2022 Apr 22;:]. Eur J Pediatr. 2022;181(6):2433–2438. 10.1007/s00431-022-04387-x. 10.1007/s00431-022-04387-x PMC8929282 35302178

[12] Cercone DJ, Berger RP, Manole MD, Soung JK, Coombs CM, Noorbakhsh KA. Increased severity of abusive head trauma during the first year of the COVID-19 pandemic. Child Abuse Negl. 2023;135:105971. 10.1016/j.chiabu.2022.105971. 10.1016/j.chiabu.2022.105971 PMC9676164 36427395

[13] Chung G, Lanier P, Wong PYJ (2022). Mediating Effects of Parental Stress on Harsh Parenting and Parent-Child Relationship during Coronavirus (COVID-19) Pandemic in Singapore. J Fam Violence.

[14] • Focardi M, Grassi S, Raddi S, et al. Trend in 167 cases of minors witnessing violence: The role played by COVID-19 pandemic. Front Pediatr. 2022;10:949922. Published 2022 Oct 5. 10.3389/fped.2022.949922. **This study highlights the institutional role in dealing with interparental IPV witnessing cases.** 10.3389/fped.2022.949922 PMC9581130 36275057

[15] •• Liu J, Chai L, Zhu H, Han Z. COVID-19 impacts and adolescent suicide: The mediating roles of child abuse and mental health conditions. Child Abuse Negl. 2023;138:106076. 10.1016/j.chiabu.2023.106076. **This study reveals the hightened impact of the pandemic and the risk of suicide ideation and behaviour.** 10.1016/j.chiabu.2023.106076 PMC9894761 36764172

[16] Massiot L, Launay E, Fleury J, et al. Impact of COVID-19 pandemic on child abuse and neglect: A cross-sectional study in a French Child Advocacy Center. Child Abuse Negl. 2022;130(Pt 1):105443. 10.1016/j.chiabu.2021.105443. 10.1016/j.chiabu.2021.105443 PMC9741962 34952733

[17] • Pannizzotto S, Depuis Z, Frère J, Seghaye MC. Face à la COVID-19.Impact de la pandémie COVID-19 sur les maltraitances intrafamiliales dans la population pédiatrique [Impact of COVID-19 outbreak on domestic violence and child abuse]. Rev Med Liege. 2021;76(11):789–793. **This study has many limitations though it revealed the institutional role in dealing with suspected cases of sexual VAC.** 34738751

[18] •• Sethuraman U, Kannikeswaran N, Singer A, et al. Trauma Visits to a Pediatric Emergency Department During the COVID-19 Quarantine and "Stay at Home" Period [published online ahead of print, 2021 Nov 16]. Am Surg. 2021;31348211047497. 10.1177/00031348211047497. **This study has many limitations though it revealed a high mortality rate due to suspected AHT.** 10.1177/00031348211047497 34784788

[19] • Sinko L, He Y, Kishton R, Ortiz R, Jacobs L, Fingerman M. "The Stay at Home Order is Causing Things to Get Heated Up": Family Conflict Dynamics During COVID-19 From The Perspectives of Youth Calling a National Child Abuse Hotline. J Fam Violence. 2022;37(5):837–846. 10.1007/s10896-021-00290-5. **This study highlighted a control tactic that might have been used against children by their parents, i.e., technological isolation.** 10.1007/s10896-021-00290-5 PMC8186368 34121803

[20] • Tso WWY, Chan KL, Lee TMC, et al. Mental health & maltreatment risk of children with special educational needs during COVID-19. Child Abuse Negl. 2022;130(Pt 1):105457. 10.1016/j.chiabu.2021.105457. **This study is one of the studies on vulnerable children during the pandemic.** 10.1016/j.chiabu.2021.105457 PMC8743505 35033372

[21] • Whaling KM, Der Sarkissian A, Larez N, Sharkey JD, Allen MA, Nylund-Gibson K. Child Maltreatment Prevention Service Cases are Significantly Reduced During the COVID-19 Pandemic: A Longitudinal Investigation Into Unintended Consequences of Quarantine. Child Maltreat. 2023;28(1):34–41. 10.1177/10775595211051318. **This study revealed the important role played by prevention service cases in timely interventions which could prevent further violence and household removals.** 10.1177/10775595211051318 PMC9805920 34908497

[22] •• Yukich J, Worges M, Gage AJ, et al. Projecting the Impact of the COVID-19 Pandemic on Child Marriage. J Adolesc Health. 2021;69(6S):S23-S30. 10.1016/j.jadohealth.2021.07.037. **This study gave much needed insight into the impact of the pandemic on an issue that is increasingly putting the lives of children in danger, i.e., child marriage.** 10.1016/j.jadohealth.2021.07.037 PMC8610224 34809896

[23] UNFP (United Nations Population Fund). Child marriage in the context of COVID-19: Analysis of trends, programming and alternative approaches in the Middle East and North Africa. 2021.

[24] Child Welfare Information Gateway. Mandatory reporters of child abuse and neglect. U.S. Department of Health and Human Services, Administration for Children and Families, Children’s Bureau. 2019.

[25] Racine N, Hartwick C, Collin-Vézina D, Madigan S. Telemental health for child trauma treatment during and post-COVID-19: Limitations and considerations. Child Abuse Negl. 2020;110(Pt 2):104698. 10.1016/j.chiabu.2020.104698. 10.1016/j.chiabu.2020.104698 PMC7437482 32839022

[26] Kolivoski KM, Shook JJ, Kim KH, Goodkind S (2017). Placement type matters: Placement experiences in relation to justice system involvement among child welfare-involved youth and young adults. J Hum Behav Soc Environ.

[27] Geprägs A, Bürgin D, Fegert JM, Brähler E, Clemens V. Parental stress and physical violence against children during the second year of the COVID-19 pandemic: results of a population-based survey in Germany. Child Adolesc Psychiatry Ment Health. 2023;17(1):25. Published 2023 Feb 20. 10.1186/s13034-023-00571-5. 10.1186/s13034-023-00571-5 PMC9940081 36804027

[28] Thomas EY, Anurudran A, Robb K, Burke TF. Spotlight on child abuse and neglect response in the time of COVID-19. The Lancet Public Health. 2020;5(7):e371. 10.1016/S2468-2667(20)30143-2. 10.1016/S2468-2667(20)30143-2 PMC7326432 32619538

[29] Fish JN, McInroy LB, Paceley MS, Williams ND, Henderson S, Levine DS, Edsall RN (2020). “I'm Kinda Stuck at Home With Unsupportive Parents Right Now”: LGBTQ Youths' Experiences With COVID-19 and the Importance of Online Support. J Adolesc Health.

[30] Walker K, Sleath E, Tramontano C. The Prevalence and Typologies of Controlling Behaviors in a General Population Sample. J Interpers Violence. 2021;36(1–2):NP474-NP503. 10.1177/0886260517731785. 10.1177/0886260517731785 29294941

[31] Carnevale S, di Napoli I, Esposito C, Arcidiacono C, Procentese F (2020). Children witnessing domestic violence in the voice of health and social professionals dealing with contrasting gender violence. Int J Environ Res Public Health.

[32] Steketee M, Aussems C, Marshall IH. Exploring the impact of child maltreatment and interparental violence on violent delinquency in an international sample. J Interpers Violence. 2021;36:NP7319–49. 10.1177/0886260518823291. 10.1177/0886260518823291 30678540

[33] McTavish JR, MacGregor JCD, Wathen CN, MacMillan HL (2016). Children’s exposure to intimate partner violence: an overview. Int Rev Psychiatry.

[34] Anastasia F, Wiel LC, Giangreco M, Morabito G, Romito P, Amaddeo A (2022). Prevalence of children witnessed violence in a pediatric emergency department. Eur J Pediatr.

[35] Council of Europe. Convention on preventing and combating violence against women and domestic violence (Istanbul Convention). 2019. https://www.coe.int/en/web/gender-matters/council-of-europe-convention-on-preventing-and-combating-violence-against-women-and-domestic-violence.

[36] Roberts AL, Gilman SE, Fitzmaurice G, Decker MR, Koenen KC (2010). Witness of intimate partner violence in childhood and perpetration of intimate partner violence in adulthood. Epidemiology.

[37] Brook C. Evidence for significant misdiagnosis of abusive head trauma in pediBIRN data. Forensic Sci Int Synerg. 2023;6:100314. 10.1016/j.fsisyn.2023.100314. 10.1016/j.fsisyn.2023.100314 PMC9860097 36691664

[38] Augusti EM, Myhre MC, Wentzel-Larsen T, Hafstad GS. Violence and sexual abuse rates before and during the Covid-19 pandemic: A prospective population-based study on Norwegian youth. Child Abuse Negl. 2023;136:106023. 10.1016/j.chiabu.2023.106023. 10.1016/j.chiabu.2023.106023 PMC9825257 36628828

[39] Chan KL, Emery CR, Ip P (2016). Children with disability are more at risk of violence victimization: Evidence from a study of school-aged Chinese children. J Interpers Violence.

[40] Scherer N, Verhey I, Kuper H. Depression and anxiety in parents of children with intellectual and developmental disabilities: A systematic review and meta-analysis. PLoS One, 2019;14(7), Article e0219888. 10.1371/journal.pone.0219888. 10.1371/journal.pone.0219888 PMC6667144 31361768

[41] McFayden TC, Breaux R, Bertollo JR, Cummings K, Ollendick TH (2021). COVID-19 remote learning experiences of youth with neurodevelopmental disorders in rural Appalachia. J Rural Ment Health.

[42] Pereda N, Díaz-Faes DA. Family violence against children in the wake of COVID-19 pandemic: a review of current perspectives and risk factors. Child Adolesc Psychiatry Ment Health. 2020;14:40. Published 2020 Oct 20. 10.1186/s13034-020-00347-1. 10.1186/s13034-020-00347-1 PMC7573863 33088340

[43] Finkelhor D, Asdigian NL (1996). Risk factors for youth victimization: beyond a lifestyles/routine activities theory approach. Violence Vict.

[44] Aneshensel CS (1992). Social stress: theory and research. Ann Rev Sociol.

[45] UNICEF. Understanding the Relationship between Child Marriage and Female Genital Mutilation: A statistical overview of their co-occurrence and risk factors, UNICEF Data and Analytics, 2021.

[46] United Nations Population Fund. Motherhood in childhood: facing the challenge of adolescent pregnancy. New York; 2013. Retrieved from https://www.unfpa.org/sites/default/files/pub-pdf/EN-SWOP2013.pdf.

[47] Misunas C, Gastón CM, Cappa C. Child marriage among boys in high-prevalence countries: an analysis of sexual and reproductive health outcomes. BMC Int Health Hum Rights. 2019;19(1):25. Published 2019 Aug 16. 10.1186/s12914-019-0212-8. 10.1186/s12914-019-0212-8 PMC6697949 31420012

[48] Foreign and common wealth office. Forced Marriage Unit Statistics 2018, UK. available at https://assets.publishing.service.gov.uk/government/uploads/system/uploads/attachment_data/file/882532/Forced_Marriage_Unit_Statistics_2018_revised_final.pdf.

[49] UNFPA-UNICEF Global Programme to end Child marriage. 2020. ‘Global Consultation on Child Marriage in Humanitarian Settings.’ Meeting Report. 4–5 February 2020, Amman

[50] Baron EJ, Goldstein EG, Wallace CT. Suffering in silence: How COVID-19 school closures inhibit the reporting of child maltreatment. J Public Econ. 2020;190:104258. 10.1016/j.jpubeco.2020.104258. 10.1016/j.jpubeco.2020.104258 PMC7441889 32863462

[51] Kavemann B (2017). Violence in Intimate Relationships - Effects on Children and Adolescents - Effects on the Mother-Child-Relationship.

[52] Steine IM, Winje D, Skogen JC (2017). Posttraumatic symptom profiles among adult survivors of childhood sexual abuse: A longitudinal study. Child Abuse Negl.

[53] Falling Through the Cracks: How Laws Allow Child Marriage to Happen in Today’s America, 2017, available at http://www.tahirih.org/pubs/falling-through-the-cracks-how-laws-allow-childmarriage-to-happen-in-todays-america/.

